# Inherited Risk Factors of Thromboembolic Events in Patients with Primary Nephrotic Syndrome

**DOI:** 10.3390/medicina56050242

**Published:** 2020-05-19

**Authors:** Gener Ismail, Bogdan Obrișcă, Roxana Jurubiță, Andreea Andronesi, Bogdan Sorohan, Mihai Hârza

**Affiliations:** 1 Department of Nephrology, Fundeni Clinical Institute, Bucharest 022328, Romania; gener732000@yahoo.com (G.I.); roxana_jurubita@yahoo.com (R.J.); andreea.andronesi@yahoo.com (A.A.); bogdan.sorohan@yahoo.com (B.S.); 2 Department of Uronephrology, “Carol Davila” University of Medicine and Pharmacy, Bucharest 020021, Romania; mihai.harza@gmail.com; 3 Center of Uronephrology and Renal Transplantation, Fundeni Clinical Institute, Bucharest 022328, Romania

**Keywords:** nephrotic syndrome, thrombosis, inherited risk factors, mutation, anticoagulation

## Abstract

*Background and objectives.* Venous thromboembolic events (VTEs) are among the most important complications of nephrotic syndrome (NS). We conducted a study that aimed to determine the prevalence of inherited risk factors for VTE in NS and to identify which factors are independent predictors of VTE. *Materials and Methods.* Thirty-six consecutive patients with primary NS that underwent percutaneous kidney biopsy between January 2017 and December 2017 were enrolled in this retrospective, observational study. VTEs were the primary outcome. Baseline demographic and biochemical data were collected from medical records, and genetic testing was done for polymorphisms of Factor V, PAI, MTHFR, and prothrombin genes. *Results.* The incidence of VTE was 28%, and the median time to event was 3 months (IQR: 2–9). The prevalence of inherited risk factors was 14% for Factor V Leiden mutation, 5.6% for prothrombin G20210A, 44.5% for PAI, and 27.8% for each of the two polymorphisms of the MTHFR gene. On multivariate analysis, the presence of at least two mutations was independently associated with the risk of VTE (HR, 8.92; 95% confidence interval, CI: 1.001 to 79.58, *p* = 0,05). *Conclusions.* These findings suggest that genetic testing for inherited thrombophilia in NS could play an important role in detecting high-risk patients that warrant prophylactic anticoagulation.

## 1. Introduction

Venous thromboembolic events (VTEs) are serious complications of nephrotic syndrome (NS), associated with significant morbidity and mortality [1]. The reported incidence varies widely (5–50%), due primarily to the retrospective nature of most studies and the lack of an accurate screening method that eludes an important number of asymptomatic cases [1,2,3,4]. Current clinical guidelines have inadequate evidence to support prophylactic anticoagulation, taking into account only serum albumin level and proteinuria over 24 h as markers of VTE risk [5].

VTEs must be viewed as a multifactorial disorder with the underlying pathogenesis being a complex interplay of inherited and acquired risk factors [6]. Hemostasis imbalance secondary to the NS [7], chronic inflammation associated with certain glomerulonephritis [1], a genetic background [1,6], when associated, can trigger a VTE. Usually, an inherited thrombophilia does not result in a spontaneous VTE, until an acquired hypercoagulable state (nephrotic syndrome) determines the clinical expression of the prothrombotic tendency [6]. With past clinical trials focusing mainly on serum albumin or proteinuria to define the VTE risk [3,8,9,10], the magnitude of additional risk factors has been evaluated only in small series, with conflicting results [2,11,12,13,14]. Additionally, polymorphisms associated with inherited thrombophilia seem to be relatively prevalent in the general population, and screening for these disorders remains debatable [2,6,15,16,17].

We conducted a retrospective, observational study that sought to identify the prevalence of inherited risk factors for VTEs in a population of NS patients and to define the risk of VTEs in such patients.

## 2. Materials and Methods

### 2.1. Study Patients’ Characteristics

All consecutive patients with primary NS admitted to our department between January 2017 and December 2017 were considered for inclusion, and only those with a histopathological diagnosis were further included in the study. NS was defined as a level of proteinuria over 3.5 g/day and hypoalbuminemia. Exclusion criteria were age under 18 years, presence of known secondary causes of NS (diabetes, hepatitis B or C virus infection, HIV infection, systemic lupus erythematosus), a previous VTE, disorders that interfere with the synthesis of hemostasis-related factors (severe hepatic impairment) and therapy that influences hemostasis (antiplatelet drugs, anticoagulants).

Baseline demographics and biochemical data collected included age at presentation, gender, proteinuria over 24 h, serum albumin, and creatinine level. Glomerular filtration rate (GFR) was estimated using the Chronic Kidney Disease Epidemiology Collaboration (CKD-EPI) equation. The genetic testing panel included the assessment for polymorphisms of Factor V gene (G1691A, T1250C, Cambridge and Hong-Kong mutations), PAI gene (plasminogen activator inhibitor—4G/5G mutation), methylene tetrahydrofolate reductase (MTHFR) gene (C677T and A1298C mutations) and prothrombin gene (G20210A mutation). Genetic testing was performed using a real-time polymerase chain reaction method (RT-PCR).

The study was conducted in accordance with the Declaration of Helsinki, and the protocol was approved by the Ethics Committee of Fundeni Clinical Institute (No. 20638/30 March 2020).

### 2.2. Diagnosis of Venous Thromboembolic Events

VTEs were suspected on clinical grounds (unilateral limb pain or edema, cough, dyspnea, hemoptysis, loin pain, hematuria) and, additionally, were screened by D-dimers level to identify asymptomatic events. The D-dimers level was determined every 3 months and considered elevated if the level was over 2 mcg/mL. VTEs were confirmed by imaging studies: deep vein thrombosis (DVT) by Doppler ultrasound, renal vein thrombosis (RVT) by Doppler ultrasound followed by contrast spiral computed tomography (CT), pulmonary embolism (PE) by spiral CT. The ultrasonographic criteria for diagnosis of venous thrombosis (deep vein, renal vein) were direct visualization of thrombi and absent venous flow. Additionally, in the case of RVT, a confirmatory CT scan was undertaken to document filling defects during the venous phase following intravenous contrast.

### 2.3. Statistical Analysis

Data distribution was evaluated with the Jurque–Bera test. Normally distributed variables were expressed as means and standard deviations, while the non-normally distributed variables were expressed as a medians and interquartile ranges (IQRs). Categorical variables were expressed as numbers and frequencies. For continuous data, differences between groups were assessed using the Student *t*-test and univariate ANOVA, depending on the level of the independent variable. For categorical data, differences between groups were evaluated using the χ^2^ test. To determine the relationship between the studied parameters and the primary endpoint (occurrence of VTE), univariate and multivariate Cox proportional hazard regression was performed. All p values were two-tailed, and all p values less than 0.05 were considered statistically significant. The time to event was measured from the baseline to the moment of documented VTE. The probability of event-free survival was assessed using a Kaplan–Meyer curve. The statistical analysis was performed using SPSS 17.0 (SPSS Inc., Chicago, IL, USA).

## 3. Results

### 3.1. Patient Characteristics

The study cohort included 36 patients with the baseline characteristics shown in Table 1. Four patients were excluded from the study due to the absence of histopathological diagnosis or identification of a secondary cause for the nephrotic syndrome (amyloidosis). Membranous nephropathy (MN) was the most common type of glomerulopathy found on kidney biopsy (38.9%), followed by IgA nephropathy (IgAN) (27.8%), minimal-change disease (MCD) (19.4%) and focal and segmental glomerulosclerosis (FSGS) (13.9%).

The baseline 24-h proteinuria and serum albumin were 7.29 ± 2.4 g/day and 2.58 ± 0.62 g/dL, respectively. Additionally, 11 patients (30.5%) were over 50 years old, while three of them were over 65 years old. The average 24-h proteinuria differed across the four types of glomerulopathies (*p* = 0.002). Patients with minimal-change disease (MCD) had higher proteinuria levels than those with membranous nephropathy (*p* < 0.001) and IgA nephropathy (*p* = 0.02), respectively. Additionally, all patients with MCD had severe nephrotic syndrome with proteinuria levels over 8 g/day. The mean baseline estimated glomerular filtration rate (eGFR) was 66.2 ± 32.7 mL/min/1.73 m^2^, and although MCD and IgA nephropathy patients seemed to have lower eGFR, it did not reach statistical significance (*p* = 0.44). The median follow-up time was 24 months (IQR 9–36). In terms of risk factors for VTE, five patients (13.9%) were obese, four patients (11.1%) were current or previous smokers, one patient had a previous history of VTE, and one patient had a diagnosis of antiphospholipid syndrome. Moreover, two patients had a history of other autoimmune disorders (ulcerative colitis and ankylosing spondylitis). There was no family history of VTE in the study cohort.

**Figure 1:**
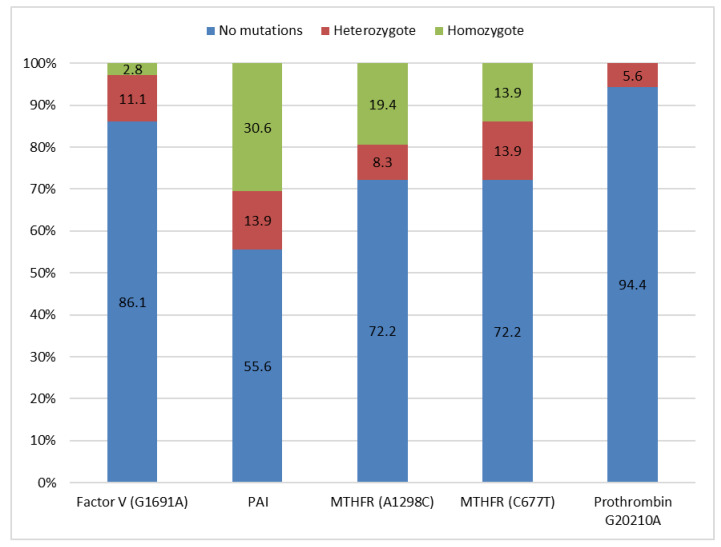
Prevalence of the inherited risk factors for VTEs in the study cohort.

The prevalence of the inherited risk factors for VTE is depicted in Figure 1. Almost 3% of the patients were homozygous and 11% heterozygous for the Factor V mutation G1691A, while none had the Cambridge, Hong-Kong, or T1250C mutations. The prothrombin G20210A mutation was encountered in 5.6% of the patients. The two polymorphisms of the methylene tetrahydrofolate reductase gene and the mutation of the PAI gene were highly prevalent in the study cohort (28% and 45%, respectively). Additionally, 12 patients (33%) had at least two of the studied mutations (Table 2).

### 3.2. Venous Thromboembolic Events: Frequency

Ten VTEs were diagnosed in the study population (28%). The median time to VTE was 3 months (IQR 2–9), with 80% and 100% of VTE occurring during the first 6 months and the first year of follow-up, respectively. The vast majority of the thrombotic events were unilateral RVT (70%), followed by bilateral RVT, DVT, and PE, each being diagnosed in one patient. The majority of VTEs were asymptomatic, except in the cases of PE, DVT, and bilateral RVT. The asymptomatic events (unilateral RVT) were diagnosed by a confirmatory imaging method following the suspicion triggered by an increase in D-dimers level. There were no significant differences in terms of VTE frequency across the different types of glomerulopathies (*p* = 0.827).

### 3.3. Venous Thromboembolic Events: Risk Factors

**Figure 2:**
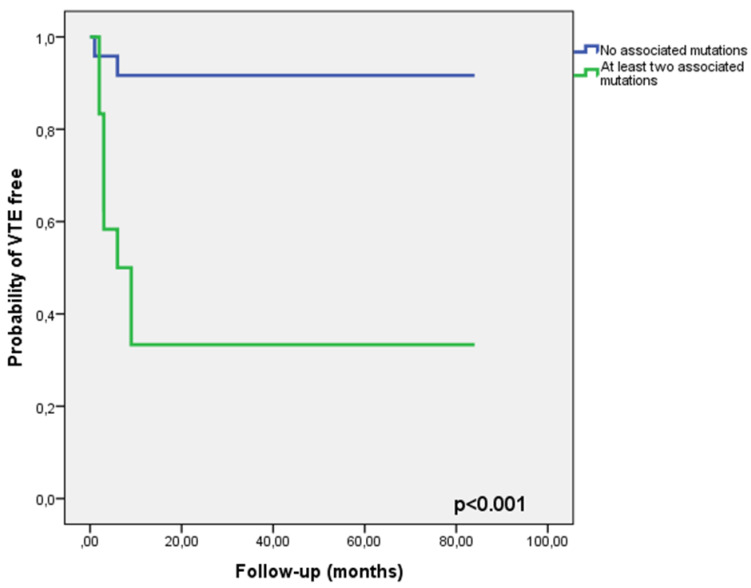
The risk of VTEs over time in patients with at least two associated mutations.

The results of the univariate analysis in order to identify clinical and biochemical risk factors for the development of VTE are shown in Table 3. Patients that developed a VTE had higher levels of proteinuria (*p* = 0.045) and lower serum albumin (*p* = 0.002) than those event-free. Additionally, patients that developed a VTE had more frequent polymorphisms of the Factor V gene (G1691A), MTHFR gene (C677T), or the 4G/5G mutation of PAI gene than those event-free. Moreover, the association of at least two of the abovementioned mutations was encountered more frequently in those that developed a VTE. The percentage of patients with obesity (*p* > 0.99), that were older than 50 years of age (*p* = 0.45) or that were current or previous smokers (*p* > 0.99) did not differ between those that developed a VTE or not. Additionally, the patients with a previous history of VTE developed a pulmonary embolism, while the patients with a previous diagnosis of antiphospholipid syndrome developed an asymptomatic unilateral renal vein thrombosis. On multivariate Cox proportional hazard regression, only the association of at least two mutations was independently associated with the risk of VTE (HR, 8.92; 95% confidence interval, CI: 1.001 to 79.58, *p* = 0.05) (Table 4), these patients having a significantly lower probability of remaining VTE-free than those with no associated mutations (*p* < 0.001) (Figure 2).

## 4. Discussion

VTEs are an important cause of morbidity and mortality among patients with nephrotic syndrome [18], with an incidence rate that varies greatly between different clinical trials [1]. Our study confirms the high prevalence of VTE (28%) in patients with primary nephrotic syndrome, with the vast majority of VTE being unilateral RVT (70%). The large variability in terms of VTE prevalence is mainly due to differences in methods used to diagnose VTE [1]. Although the frequency distribution of VTE appears to have changed over time—past clinical trials report RVT as the most common type of VTE [1,19,20,21], while more recent papers show a higher prevalence of PE and DVT [7,10]—caution must be employed when interpreting these results. Studies conducted in the 1980s and 1990s used renal venography as a screening method for RVT [19,20,21,22], capturing many asymptomatic events and thereby accounting for the higher prevalence of RVT, while the more recent retrospective studies focused mainly on symptomatic events [2,9,10]. Chronic RVT is usually asymptomatic and tends to occur more in older patients (mean age 40 years) than acute RVT (mean age 20 years) [1,2]. Our study population had a mean age of 43.4 ± 14.2 years, and we used a screening method for VTE detection (D-dimers levels), thereby explaining the higher prevalence of RVT in our cohort. The significance of screening asymptomatic events is debatable, but the presence of asymptomatic RVT determines an increased risk of PE, and a screening method could be undertaken at least in high-risk patients [23]. The predilection for RVT in nephrotic syndrome, and especially in MN, supposedly involves local disturbances in hemostasis, with generation of thrombin, and fibrinolysis, but this remains unproven [1,2,19].

Currently, the risk assessment of VTE in NS relies mainly on serum albumin, proteinuria level, and histological diagnosis [2,5]. Controversy exists regarding the cut-off limit for serum albumin, below which a high VTE risk could be defined [2,3]. Although most studies identified hypoalbuminemia (serum albumin below 2.5 g/dL) as an independent risk factor for VTE [3,7,8,9,20], others did not show significant differences between patients with or without VTE [3,21]. Our study did not identify serum albumin or proteinuria as independent risk factors for VTE. Despite the fact that most of our patients had severe hypoalbuminemia, we detected VTE even in patients with only a modest reduction in serum albumin (3 g/dL). As previously stated by other papers [2,3], it seems that hypoalbuminemia is a risk factor for VTE, but it is not mandatory for the development of these complications. In terms of histopathological diagnosis, MN is universally recognized as being associated with the highest risk for VTE development [2,8,24], although other studies did not confirm this predilection [10]. 

Uncertainties exist about which patients with idiopathic GN, other than MN, or with less severe NS would benefit from prophylactic anticoagulation, and identification of these high-risk patients still remains a challenge. The pathogenesis of thromboembolic complications in NS is multifactorial, with a complex interplay of acquired and inherited factors [1,6]. Although hemostasis derangements with a shift towards a prothrombotic state is well recognized in NS, studies linking these disturbances to VTE development are somewhat lacking [1]. Antithrombin III (AT III) deficiency is encountered in up to 80% of patients with NS [3]. Despite the fact that AT III deficiency reflects the severity of NS, as it is being correlated with the degree of hypoalbuminemia and proteinuria, not all studies confirmed the association between AT III deficiency and VTE [3]. Previous work of our group identified ionized calcium and a lower AT III activity as being independent predictors of VTE [7].

While most studies over the past decades evaluated mainly acquired risk factors for VTE in NS (serum albumin, proteinuria, hemostasis parameters) [8,9,10], few have addressed inherited risk factors with conflicting results [2,11,12,13,14]. 

Genetic mutations associated with inherited thrombophilia are frequently encountered in the general population [2,6,16,17,25,26,27]. Despite that the presence of these polymorphisms determines from a 3-fold increased risk (for prothrombin G20210A) to up to an 80-fold increased risk (for factor V Leiden homozygote) [6], the incidence of thrombotic events in susceptible individuals is highly variable, with many of them never developing VTE [17]. Although our study enrolled consecutive patients, we identified a higher prevalence of Factor V G1691A, prothrombin G20210A, and MTHFR polymorphisms than those encountered in the general population [6,28]. Additionally, 33% of our patients had at least two mutations, while the reported prevalence of heterozygosity for Factor V and prothrombin is 0.1%, and other associations have not been evaluated [6]. In univariate analysis, the presence of polymorphisms for the Factor V gene (G1691A), MTHFR gene (C677T), and the 4G/5G mutation of the PAI gene, and in addition, the presence of at least two mutations was associated with VTE. In multivariate analysis, only the combination of at least two mutations was identified as an independent predictor of VTE. Many studies over the past years have evaluated the risk of initial and recurrent VTE in patients carrying a single mutation [29,30]. Although the presence of polymorphisms for Factor V and prothrombin appears to increase the risk of initial VTE, most of the studies showed similar rates of VTE recurrence among carriers and non-carriers of thrombophilic mutations [29,31]. The general agreement exists that, in this setting, screening for these thrombophilias should not be undertaken because it can misguide the clinical decision on appropriate anticoagulation treatment duration [32]. Nevertheless, patients with double heterozygosity for factor V and prothrombin had, in one study, a 2.6-fold higher risk for VTE recurrence compared to those heterozygous only for factor V [33]. Despite not being supported by all studies [31], patients with coexistent thrombophilias may warrant prolonged anticoagulation [28]. Altough based on a small number of patients, the results of this study outline several important concepts. First of all, we confirmed that VTE is a multigenic disorder by the observation that the combination of two mutations confers an increased risk of VTE, as compared to the presence of either one alone [6]. The presence of these polymorphisms must be viewed as a risk factor for VTE, whose clinical expression is determined by acquiring the hemostasis disturbances associated with the NS [17]. Taken together, an increased level of Factor V associated with NS [1] in conjunction with inherited resistance to inactivation by protein C, coexistent mutations that cause higher plasma prothrombin levels, or hyperhomocysteinemia could explain why only some patients develop VTE in the setting of similar NS severity. Despite the fact that controversy still exists regarding the risk of coexistent thrombophilias for initial and recurrent VTE, none of those studies have addressed this concern in the setting of NS, and none of them have evaluated associations other than Factor V-prothrombin [29,30,31,33,34]. Since the majority of VTE in our study occurred during the first 6 months of follow-up, thereby confirming that the highest risk is within this time frame [7,9], early identification of high-risk patients for prophylactic anticoagulation is mandatory, and screening for thrombophilic defects might be of value in this setting (Table 5). Additionally, other studies have proposed the use of monitoring the D-dimers level as a screening method for VTE [7] that could reliably predict the risk of VTE recurrence, suggesting a possible role in tailoring anticoagulation treatment in these patients [35]. However, our study has some limitations (retrospective study, a small number of patients) that prevents the generalizability of the results. As such, larger, prospective, multicenter cohorts are needed to fully validate these findings.

Therefore, in the view that thromboembolic complications of NS are multifactorial in origin [1], one could not accurately predict the risk of VTE in NS while taking into account only serum albumin and proteinuria as risk stratifying markers. A risk score that would encompass serum albumin, hemostasis parameters (such as AT III), and the presence of these polymorphisms could better stratify these patients, but it needs to be validated in large clinical trials.

## 5. Conclusions

In summary, the association of two genetic mutations confers an independent risk for VTE in NS. Therefore, genetic testing for inherited thrombophilia in NS could play an important role in detecting high-risk patients that warrant prophylactic anticoagulation.

## Figures and Tables

**Table 1:** Characteristics of the Study Population.

Variable	Overall	MN	MCD	FSGS	IgAN	*p*-Value
**Number of patients**	36	14	7	5	10	
**Number of VTEs**	10	5	2	1	2	0.827
**Age (y)**	43.4 ± 14.2	47.7 ± 14	40.5 ± 19.8	44.4 ± 12.4	39.2 ± 11	0.52
**Serum albumin (g/dL)**	2.58 ± 0.62	2.75 ± 0.52	2.2 ± 0.64	2.52 ± 1	2.66 ± 0.48	0.28
**Proteinuria (g/day)**	7.29 ± 2.4	6.2 ± 1.42	10.1 ± 0.7	7 ± 2.1	7 ± 3	0.002
**Serum creatinine (mg/dL)**	1.5 ± 0.9	1.37 ± 0.9	1.71 ± 1.2	1.07 ± 0.25	1.75 ± 0.88	0.48
**Estimated GFR (mL/min/1.73 m^2^)**	66.2 ± 32.7	75.2 ± 38.1	63.4 ± 30.1	70.8 ± 20.6	53.3 ± 30.5	0.44

Abbreviations: VTE, venous thromboembolic events; GFR, glomerular filtration rate. For continuous data, differences between groups were assessed using univariate ANOVA, and for categorical data, differences between groups were evaluated using the χ^2^ test.

**Table 2:** Type of mutations in patients with at least two associated mutations.

Patient	Factor V (G1691A)	PAI 4G/5G	MTHFR (A1298C)	MTHFR (C667T)	Prothrombin G20210A
**1**	Homozygote	-	-	-	Heterozygote
**2**	Heterozygote	Heterozygote	-	Homozygote	-
**3**	Heterozygote	Homozygote	Homozygote	-	-
**4**	-	Homozygote	Homozygote	-	-
**5**	Heterozygote	Homozygote	-	-	-
**6**	-	Homozygote	Heterozygote	Heterozygote	-
**7**	-	Homozygote	Homozygote	Homozygote	-
**8**	-	Heterozygote	-	Homozygote	-
**9**	-	Homozygote	Homozygote	Homozygote	-
**10**	-	Heterozygote	Homozygote	-	-
**11**	-	Homozygote	Homozygote	-	-
**12**	Heterozygote	Homozygote	-	-	-

Abbreviations: MTHFR, methylene tetrahydrofolate reductase; PAI, plasminogen activator inhibitor.

**Table 3:** Patients characteristics at baseline.

Parameter	With VTE	Without VTE	*p*-Value
**Age (years)**	41.9 ± 18	44 ± 12.8	0.742
**Proteinuria (g/day)**	8.62 ± 2.27	6.78 ± 2.29	0.045
**Serum Albumin (g/dL)**	2 ± 0.6	2.8 ± 0.48	0.002
**Serum creatinine (mg/dL)**	1.56 ± 0.9	1.48 ± 0.92	0.808
**eGFR (mL/min)**	66.5 ± 37.61	66.1 ± 3.44	0.978
**MTHFR (A1298C) (%)**	50%	19.2%	0.1
**MTHFR (C677T) (%)**	60%	15.4%	0.01
**Factor V (G1691A) (%)**	60%	3.8%	0.015
**Prothrombin G20210A (%)**	10%	3.8%	0.48
**PAI 4G/5G mutation (%)**	80%	30.8%	0.011
**Association of two mutations (%)**	80%	15.4%	0.001

Abbreviations: eGFR, estimated glomerular filtration rate; MTHFR, methylene tetrahydrofolate reductase; PAI, plasminogen activator inhibitor. For continuous data, differences between groups were assessed using the Student *t*-test, and for categorical data, differences between groups were evaluated using the χ^2^ test.

**Table 4:** Risk factors for venous thromboembolic events in patients with primary nephrotic syndrome (Cox proportional hazards model).

Variable	Univariate Analysis		Multivariate Analysis	
	Hazard Ratio (95% CI)	*p*-Value	Hazard Ratio (95% CI)	*p*-Value
**Serum Albumin (for each 1 g/dL)**	0.25 (0.11–0.58)	0.001	0.43 (0.1–1.89)	0.27
**24-h proteinuria (for each 1 g/day)**	1.33 (1.009–1.75)	0.04	1.14 (0.79–1.64)	0.46
**MTHFR (A1298C) (presence vs. absence)**	2.91 (0.84–10.11)	0.09	0.49 (0.07–3.11)	0.45
**MTHFR (C677T) (presence vs. absence)**	4.51 (1.27–16.02)	0.02	1.38 (0.23–8.36)	0.72
**Factor V (G1691A) (presence vs. absence)**	6.4 (1.77–23.08)	0.005	0.92 (0.12–6.83)	0.94
**Prothrombin G20210A (presence vs. absence)**	2.68 (0.33–21.38)	0.35	3.23 (0.26–39.9)	0.36
**Association of two mutations (presence vs. absence)**	10.51 (2.21–49.92)	0.003	8.92 (1.001–79.58)	0.05

Abbreviations: MTHFR, methylene tetrahydrofolate reductase.

**Table 5:** Evaluation of the risk factors for VTE in patients with NS.

**History**	Age, smoking history, previous or family history of VTE, pregnancy, prolonged immobilization, surgery, review of concomitant medication, neoplasia, chronic heart of pulmonary disorders, presence of central venous catheters, presence of inflammatory conditions
**Laboratory predictors of VTE**	Serum albumin level, 24-h proteinuria, D-dimers level, complete blood cell count, serum ionized calcium
**Hemostasis-related protein disturbances**	Coagulation parameters (prothrombin time, activated partial thromboplastin time, serum fibrinogen, antithrombin III, protein C and S, assessment of individual coagulation and fibrinolytic factors)
**Genetic background**	Protein C and S deficiency, antithrombin III deficiency, screening for polymorphisms of Factor V gene, PAI gene, methylene tetrahydrofolate reductase (MTHFR) gene and prothrombin gene (G20210A mutation).
**Other inherited or acquired hypercoagulable states**	Antiphospholipid syndrome (lupus anticoagulant, anticardiolipin antibodies, anti-β2-glycoprotein 1 antibodies), screening for other autoimmune or connective tissue disorders associated with an increased risk for VTE depending on the clinical scenario (e.g., inflammatory bowel disease).
